# Stimulation of cGAS-STING pathway as a challenge in the treatment of small cell lung cancer: a feasible strategy?

**DOI:** 10.1038/s41416-024-02821-5

**Published:** 2024-08-31

**Authors:** Giulia Miglietta, Marco Russo, Giovanni Capranico, Jessica Marinello

**Affiliations:** Department of Pharmacy and Biotechnology, Alma Mater Studiorum—University of Bologna, Bologna, Italy

**Keywords:** Small-cell lung cancer, Immune evasion

## Abstract

Lung cancer has a significant incidence among the population and, unfortunately, has an unfavourable prognosis in most cases. The World Health Organization (WHO) classifies lung tumours into two subtypes based on their phenotype: the Non-Small Cell Lung Cancer (NSCLC) and the Small Cell Lung Cancer (SCLC). SCLC treatment, despite advances in chemotherapy and radiotherapy, is often unsuccessful for cancer recurrence highlighting the need to develop novel therapeutic strategies. In this review, we describe the genetic landscape and tumour microenvironment that characterize the pathological processes of SCLC and how they are responsible for tumour immune evasion. The immunosuppressive mechanisms engaged in SCLC are critical factors to understand the failure of immunotherapy in SCLC and, conversely, suggest that new signalling pathways, such as cGAS/STING, should be investigated as possible targets to stimulate an innate immune response in this subtype of lung cancer. The full comprehension of the innate immunity of cancer cells is thus crucial to open new challenges for successful immunotherapy in treating SCLC and improving patient outcomes.

## Introduction

Lung cancer is the most common type of cancer (12.4% of all cases) and the leading cause of cancer death [1]. One of the main risk factors for the onset of the disease is tobacco exposure, with some subtypes appearing exclusively in heavy smokers [2]. It is essentially classified into two distinct categories: Non-Small Cell Lung Cancer (NSCLC) and Small Cell Lung Cancer (SCLC). The latter is the most aggressive subtype among all lung cancers and accounts for about 15% of all cases [3]. It has predominantly neuroendocrine features, with rapid growth and metastases to distant sites in the body (brain and liver) [4]. In fact, only one-third of patients are diagnosed in the so-called limited stage of disease (LD), while the other patients have already metastases at the time of diagnosis (extensive stage of disease, ED) with consequently lower chances of survival [5]. The genetic landscape and tumour microenvironment identify SCLC as non-immunological responsive tumours [6]. This review illustrates all the various aspects related to possible improved immunotherapy, specifically addressing the stimulation of the cGAS/STING pathway as feasible pharmacological target for stimulating innate immunity as well as transforming the cold tumour phenotype. Thus, the aim of this study is to evaluate whether these approaches can actually translate into increased patient survival.

## The genetic landscape of small cell lung cancer

SCLC has a high rate of mutations, probably related to exposure to mutagens of smoke [7]. Mutation patterns in many cases are specific for each patient although some are generally conserved. About 90% of patients with SCLC have a bi-allelic inactivation mutation in the DNA binding domain of TP53, which affects the function of the protein [8]. TP53 is an important player in maintaining genome integrity and induces apoptosis and cell cycle arrest in the presence of genomic stress. *TP53* mutation appears to be an early event during SCLC evolution leading to gene instability and subsequent mutations in other tumour suppressor genes such as *RB1*, the most commonly mutated gene along with *TP53* (co-mutation rate up to 90%) [9]. Further confirming the essential role of TP53 in tumorigenesis, it has recently been reported that some patients with wild-type TP53 but possessing a specific partial deletion in the *TP73* gene, exhibit a phenotype of a dominant-negative protein function toward TP53 [9]. Downregulation of TP53 has a strong impact on many regulated proteins, such as BCL2, which is commonly upregulated in SCLC and results in escape from apoptosis [10]. Similar to TP53, RB1 is able to regulate cell cycle progression and suppresses G1-S transition under stress conditions. Loss of RB1 in SCLC results in increased cell plasticity, enabling phenotypic switch and reprogramming. Its mutation is mainly associated with the de-repression of pluripotency genes (*OCT4* and *SOX2*) and increased expression of *EZH2*. Dual inactivation of *TP53* and *RB1* in mice is a widely used procedure to develop in vivo cancer models similar to human SCLC [11].

Additional mutations are often present in SCLC, although less represented. Somatic copy number alteration analysis revealed a mutually exclusive, focal gene amplification of *MYC*, *MYCN* or *MYCL* in 16% of the studied cohort [12, 13]. Inactivating mutations of the tumour suppressor gene *PTEN*, resulting in activation of the oncogenic PI3K pathway, have been reported in different studies [12, 14–16]. Mutations in epigenetic regulators (*CREBBP*, *EP300*, *MLL*, *MLL2* and *EZH2*) and in the NOTCH family (mostly *NOTCH1*) are also present in SCLC, with the latter having a main impact on neuroendocrine differentiation and expansion [9, 17]. Despite all this genetic information already obtained, further analysis and genotyping of patients may be pursued to allow patient stratification and the development of personalised therapy.

SCLC can be divided into neuroendocrine (NE subtype) and non-neuroendocrine (non-NE subtype) [9, 18]. SCLC characterised by NE differentiation highly express transcriptional regulators *ASCL1* and *NEUROD1* and shows classic neuroendocrine morphology with in vitro cells growing in non-adherent tumour clusters [19]. Non-NE SCLCs account for about 15% of all SCLCs, upregulate transcription factors such as *POU2F3* and *YAP1*, and show adherent in vitro growth [19]. Recently, the classification of SCLC into four groups based on the expression of the genes just mentioned has been proposed (NAPY) [20], and it has been shown that subtype may drive drug sensitivity [21]. Indeed, multi-omic data obtained from 118 SCLC cell lines (SCLC CellMiner database) showed a strong correlation between drug sensitivity profiles and transcriptional networks [22]. In this study, *Schlafen11* expression was found to be the most significant genomic predictor for the NE subtype, with about 40% of SCLC cell lines not expressing the gene and being resistant to DNA-damaging agent. In contrast, non-NE SCLC does not respond to Temozolomide-based therapies because it expresses *MGMT* gene (methylguanine methyltransferase). In addition to that, the tumour subtype may influence not only the response to standard chemotherapeutics but also to immune checkpoint inhibitors (ICB), which do not significantly increase the survival of SCLC patients. Correlation between the native immune response and the expression of antigen-presenting genes of each tumour subtype indicates that only the expression of *YAP1* positively correlates to cGAS, STING, HLA-E and other interferon-inducible genes. In contrast, the expression of *NEUROD1* and *ASCL1* correlates negatively with the expression of previously mentioned immune genes [22]. The SCLC subtype expressing *YAP1* is one of the most controversial, especially considering that a very recent publication revealed that most SCLC-Y tumour cell lines are *SMARCA4* mutated, and demonstrating through a histopathological and molecular study that these tumours are strongly related to SMARCA4-deficient malignancies. In fact, SCLC-Y cells mutated in SMARCA4 are SMARCA4-deficient undifferentiated malignancies (SMARCA4-UTs), a lung tumour that mimics SCLC. Significantly, the SMARCA4-UTs show widespread expression of the YAP1 protein and its expression is a feature of many lung cancers, supporting the conclusion that it is not a reliable transcription factor for SCLC classification [23]. The identified SMARCA4-UT cell lines show a high level of MHC antigen presentation genes, suggesting that they may be immune-responsive tumours, even considering that clinical reports indicate that these tumours respond to ICB [24–26]. Recently, IHC profiling and transcriptional subtyping of primary SCLC tumours have led to the identification of a NAPY-negative and an inflamed SCLC (SCLC-I) subtypes, respectively, that share similarities in terms of response to immunotherapy [21, 27]. The plasticity and heterogeneity of SCLC is an evident feature of the tumour, and thus the SCLC classification is being debated, as discussed in the recent study by D. Shames and coworkers [28]. Using a large dataset of 271 patient samples from the IMpower133 trial, they define new tumour subsets with different intrinsic and extrinsic cell features. Considering gene expression, they indicate that *ASCL1* and *YAP1* do not uniquely define a subset; rather, both of these genes are highly expressed in more than one subset, overcoming the previous classification based on the expression of these master transcription factors. Moreover, they indicate that two of their subclusters are characterised by inflamed features and antigen presentation machinery, such as SCLC-I in the previous classification. SCLC-I had previously been characterised by low expression of *ASCL1*, but the authors now show that among these two subsets, ASCL1 is high in only one of them, suggesting that the SCLC classification should be expanded and better defined, especially considering the immune heterogeneity within the subsets [28].

## Standard therapies and immunotherapy in SCLC

Chemotherapy, possibly combined with radiation therapy, is widely used for the treatment of SCLC, and initially tumours respond well [29–31]. Unfortunately, shortly after the end of treatment, recurrence occurs in most patients and the prognosis is poor, with a two-year survival rate around 5% [32, 33]. For many decades, first-line therapy has been based on the use of cisplatin or carboplatin with etoposide or irinotecan, while after tumour relapse, topotecan is the drug of choice [31]. All the used drugs essentially belong to the category of DNA cross-linking or topoisomerase inhibitors, which affect the ability of cells to replicate DNA and the subsequent generation of double-strand breaks (DSB). Interestingly, after many years with no new approved drugs, in 2020 Lurbinectidin was added by FDA as monotherapy in patients with advanced stage of disease progression or in second-line treatment [34]. This drug is an alkylating agent and, binding preferentially to CG-rich gene promoters, it acts as a transcription inhibitor inducing RNA Pol II degradation and cell death [35, 36]. Nevertheless, the lack of viable alternative therapeutic options is one of the crucial problems in SCLC. Recently, new strategies have been directed at combining standard therapy with compounds that promote replication stress, such as inhibitors of ATR, PARP, CHK1 and WEE1, with encouraging results in clinical trials, but much still needs to be done to improve patient’s outcome [37].

Immunotherapy aims to overcome the tumour immune escape mechanism by preventing the cancer-mediated inhibition of T-cell activation and allowing reactivation and maturation of immune cells [38]. Cytotoxic T lymphocyte-associated antigen 4 (CTLA-4) was the first protein to be targeted to manipulate the immune system against cancer, as its blockade enhances the T-cell immune response and inhibits tumour growth [39]. Subsequently, anti-PD-1 antibodies were developed demonstrating lower toxicity and improved survival benefits [40]. Essentially, PD-L1 is highly expressed in many types of tumours and binds the PD-1 receptor protein expressed in T, NK and B cells, downregulating the immune response and allowing immune escape. Nivolumab was the first PD-L1 blocking antibody to be approved by FDA and although recent research advances have been promising, still only a fraction of patients benefit from immune-based therapy, and some tumours do not respond to checkpoint blockade at all. [41]. The biomarker strategy has been adopted to predict the benefits of immunotherapy. The most widely used biomarker is the PD-L1 [42]. However, the expression of this protein is a dynamic phenomenon that changes according to tumour cell interactions, tumour microenvironment, but also other factors such as previous systemic therapies, leading to inconsistent results between biomarkers and patient benefits [42].

In SCLC patients, immune checkpoint inhibitors in combination with traditional chemotherapies were recently approved. In the IMpower133 trial, the addition of anti-PD-L1 atezolizumab to the first-line chemotherapy treatment resulted in an overall survival improvement of 2 months [43, 44]. In the CASPIAN trial, another anti-PD-L, durvalumab, leaded to a similar result in terms of overall survival comparing to platinum-etoposide treatment [45]. These two compounds are recommended at the moment in first-line treatment in addition to chemotherapy [46]. Other Phase III trials that involved the use of anti-PD-L1 antibodies plus chemotherapy (adebrelimab in the CAPSTONE-1 [47] trial and serplulimab in the ASTRUM-005 trial [48]) showed a similar increase in overall survival compared to chemotherapy alone. While the increase in terms of survival in clinical trials of anti-PD-L1 compounds are significant, the actual improvement is still modest, limiting the benefit of immunotherapy in SCLC patients.

Other immunotherapy targets were explored in these years. One of these is the T-cell immunoreceptor with IG and ITIM domain (TIGIT), an immune checkpoint inhibitor that represses antitumor immune response [49]. Targeting TIGIT in combination with anti-PD-L1 showed promising results in preclinical studies [50], but Phase III trial of anti-TIGIT to anti-PD-L1+chemotherapy did not provide any benefit [51, 52]. Other promising targets in combination with anti-PD-L1 are the anti-LAG-3 monoclonal antibody therapy, that in a Phase II study met the expansion criteria in SCLC and two other tumour types [53], and Delta-like ligand 3 (DLL3), with many different ongoing Phase I/II trials with BiTE antibodies and CAR-T therapies [54–56].

While the actual immune checkpoint inhibitors line of treatment in SCLC lack of a significant improvement in survival of patients, many promising targets are being tested in clinical trials. However, despite the mutational burden of SCLC, this tumour type somehow harbours strong immunosuppressive mechanisms and represses the immune system, and these mechanisms may be potential new targets for developing new therapies.

## Immunosuppressive mechanisms in SCLC

Cancer cells are known to interact with the so-called tumour microenvironment (TME), which includes immune cells, endothelial cells and fibroblasts. TME plays a crucial role in influencing the response to ICB [28]. SCLC is characterised by a highly immunosuppressive TME that drives tumour evasion against host immune surveillance, particularly in the NE subtype [6]. In any case, the TME itself could shape the SCLC phenotype complexity and plasticity. Non-NE TME is characterised by natural killer (NK) cells, B cells and M1 tumour-associated macrophages and by the upregulation of immune co-activators, regulatory T cells and immune check points. Differently, NE and hybrid–NE subtypes correlate with a reduced intrinsic immune activation and with cancer-associated fibroblasts (CAFs), macrophages M2, matrix remodelling and pro-tumour cytokines [57]. Comprehensive studies which combine transcriptomic analysis, histopathology and proteomic data demonstrate the role of TME and the SCLC molecular phenotype in determining SCLC heterogenicity and immune cells tumour infiltration, with the final aim to better predict patient prognosis [57, 58].

**Fig. 1:**
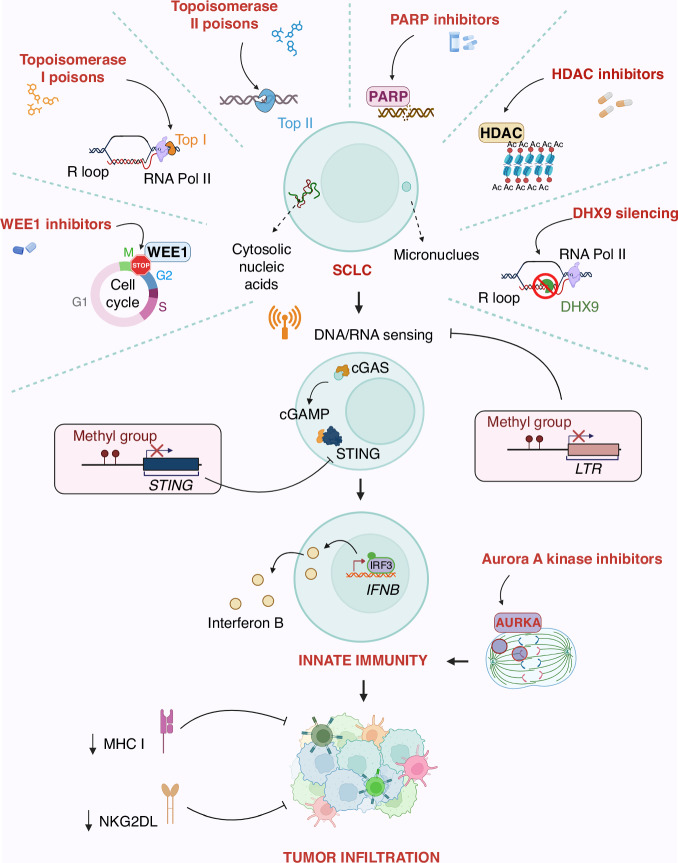
Strategies to trigger innate immune responses through cGAS/STING pathway activation in SCLC. Inhibitors of WEE1, PARP and HDAC, as well as Top I and Top II poisons and DHX9 silencing, lead to micronuclei formation and/or nucleic acid cytoplasmatic accumulation. The cytoplasmatic DNA accumulation and/or the micronuclei content in SCLC cancer cells promote the cGAS recruitment or the activation of other DNA/RNA sensors. Activation of cGAS leads to the synthesis of cGAMP, which in turn causes STING phosphorylation and its translocation to the Golgi apparatus. STING signalling promotes a transcriptional cascade of interferon-related genes critical for an innate immune response and efficacy of ICB therapy. In SCLC, the signalling could be repressed by the epigenetic regulation of LTR in SCLC and by the methylated status of the STING promoter. Inhibition of G2/M Aurora A kinase is responsible for stimulating innate immunity by a mechanism directly related to promoting immune cell infiltration within the tumour. MHC I and NKG2L low expression, caused by an epigenetic repressive mechanism, determine a reduced tumour immune cells infiltration and drive SCLC immune evasion (Created with Biorender.com, agreement number TT273EZJZ8).

Tumour immunological cold features are also associated with the alteration of STING signalling pathway in human SCLC tumours [59]. In general, in almost all cancer types, *STING* gene expression is positively correlated with immune cell infiltration and interferon response. Focusing attention on lung cancer, *STING* is significantly downregulated in SCLC compared to NSCLC (lung adenocarcinoma and lung squamous cell carcinoma) and to normal lung tissues, with a correlated downregulation of both IRF3 and NF-kB signature genes [59] (Fig. 1). Analysis of specific SCLC cell lines demonstrated that *STING* promoter shows a high methylation level that strongly affects gene expression. Through CellMiner Cross Database it has been observed that *STING* expression in lung cancer positively correlates with antigen-presenting machinery score, a prediction index for tumour response to ICB, while negatively correlates with *STING* gene methylation [59]. All these data suggest that SCLC represses *STING* expression via promoter methylation and this event may be part of a tumour escape mechanism from innate immune surveillance.

The major histocompatibility complex I (MHC I) is important to present antigen peptides to CD8^+^ T cells. The mechanism of genetic or epigenetic downregulation of *MHC-I* expression is often employed by malignant cells to escape from the immune system, similar to STING pathway downregulation [60] (Fig. 1). Most human SCLC displays low levels of MHC I, lacking therefore a crucial mechanism in the immunosurveillance system [61, 62]. In a recent study, SCLC patients were profiled and only about 15% showed high expression of MHC I. Samples classified as low or negative were rather found to have only focal clusters of tumour cells with high level of MHC I [61]. Interestingly, samples with high expression of MHC I, upregulate markers of the epithelial-to-mesenchymal transition (such as AXL) and unexpectedly downregulate ASCL1 even though they retain some neuroendocrine features. This transition of SCLC to a non-neuroendocrine/mesenchymal state demonstrates a more pronounced response to ICB and overall better survival, with tumours more infiltrated by CD45^+^/PD-L1^+^ immune cells and CD3^+^ T cells. It has also been demonstrated that the epigenetic regulator EZH2 can drive the transition to a non-neuroendocrine phenotype and its inhibition, followed by STING agonism, can be used to trigger immunogenicity in vivo by restoring MHC-I expression on the membrane [61]. In addition, SCLC lacks expression of surface NKG2DL (NKG2D ligands) favouring immune evasion mechanism [63] (Fig. 1). Indeed, the antitumor activity of the immune system depends on both T and NK cells, and the activity of the latter is strongly stimulated by NKG2DL on surface cancer cells. Restoring the expression of NKG2DL allows tumour growth suppression in SCLC mouse models [63].

Dora et al. analysed more than 200 SCLC tissues from surgically resected patients, demonstrating that about 60% can be considered immune-deserted tumours, with the TME characterised by the presence of cancer-associated fibroblasts having an immunosuppressive and tumorigenic role, albeit expressing high levels of STING [64]. In addition, although about 22% of these tumours were characterized by high levels of MHCII, an important player in the recognition of the tumour by the immune system, these patients demonstrated no survival benefits, further confirming the crucial role of the TME in effective immune cell infiltration [64].

Ongoing research attempts show promising results in the exploration of pharmacological methods involving the upregulation of transposable elements (TEs) expression, which have been shown to induce an innate immune response [65–67]. Recently, Russo et al. analysed RNA-Seq data from 104 cancer samples and 24 normal samples, showing that in SCLC tumour samples derived from patients, compared to normal ones, there is a transcriptional repression of intergenic transposable elements whose expression positively correlates with innate immune genes activation [68]. They found that this repression is likely due to the demethylase activity of LSD1, overexpressed in SCLC tumours, which causes the removal of euchromatin marker H3K4me2, suggesting that epigenetic regulation may play an important role in SCLC immunosuppression [68].

Even though SCLC are characterised by a highly immunosuppressive behaviour, it is worth noting that mutational burden, tumour subtypes and/or pharmacological treatments could contribute to activating immunological pathways, a prerequisite for effective immunotherapy. Considering a recent characterization of subtypes, it is evident that a NE subtype, which expresses *ASCL1* transcription factor, benefits of the atezolizumab treatment compared with placebo [28]. In this study, the authors indicate that despite two subtypes of immune and inflamed SCLC tumours have been defined, for which both are expected to be responsive to immunotherapy, NE tumours characterised by a low level of infiltrating macrophages are responsive to anti-PD-L1 treatment plus chemotherapy, unlike non-NE tumours with high levels of macrophages [28]. These findings suggest that a more in-depth assessment of tumour phenotype with a view to personalized therapy is clearly crucial for predicting and understanding response and resistance to current therapies and immunotherapies, especially in tumour such as SCLC in which many immune suppressive mechanisms have been described.

It has also been demonstrated that ferroptosis, together with other types of immunogenic cell death forms, can induce local inflammation and alter the tumour microenvironment, allowing infiltration of lymphocytes and somewhat increasing the immunogenicity of SCLC [69, 70]. Tumour conversion from cold to hot allows better response to ICB therapy, and has also been demonstrated by the combination of gemcitabine with a chk1 inhibitor (SRA737) along with anti-PD-L1 treatment. Specifically, the drug combination induces T cells, dendritic cells and macrophages infiltration in cancer models. It also modulates macrophage phenotype switching and activates STING and interferon signalling, with induction of CCL5 and CXCL10 transcription [71]. CCL5 is a predictive biomarker of patient response to ICB, as SCLC cancer that expresses high levels of CCL5 presents a hot TME that is associated to a longer overall survival after immune therapy [72].

Overall, although SCLC is generally an immune-unresponsive tumour, as evidenced by the many active immunosuppressive mechanisms, recent studies and more in-depth analyses suggest that there are challenges in promoting the transition from cold to hot SCLC tumours, providing a better prospect for immunotherapeutic efficacy. Moreover, the role of TME in determining the SCLC phenotype appears to be essential and provides new insights into understanding the molecular subtypes of SCLC.

## Modulation of cGAS/STING pathway to improve immunotherapy response in SCLC: a good strategy?

Target identification to enhance ICB response in SCLC appears to be the key to improving patient survival. Recently, new immunotherapeutic approaches have been proposed, mostly relying on activating the innate immune response in tumour cells. One of them concerns loss of function mutations of *NOTCH* that account for about 20–25% of SCLC tumours [73]. *NOTCH* is primarily a tumour suppressor gene as its re-expression inhibits SCLC tumour initiation. Nevertheless, it can also have a role in non-neuroendocrine plasticity, driving the cell population to a more chemo-resistant subtype [74]. In a mouse model, unlike Notch-wt cells, Notch2-mutant cells were demonstrated to be responsive to stimuli triggering the interferon response, with significantly increased total-STAT1, phospho-STAT1 and STING protein levels [74]. Indeed, both treatments with STING agonist and 2’3-cGAMP can induce CXCL10 production and block cell proliferation specifically in Notch2-mutated but not in Notch1-mutated or Notch-wt SCLC [74]. These data suggest that Notch activity represses the activation of the STING pathway. Recently it has been demonstrated that Notch signalling can upregulate the expression of *APM* genes in SCLC, particularly when a low-NE phenotype is induced, suggesting Notch signalling as a determinant of clinical benefit to immune checkpoint blockade [75].

Taniguchi et al. demonstrated that inhibition of WEE1 leads to activation of the STING-TBK1-IRF3 pathway, increased secretion of type I interferons (IFN-α and IFN-β), and pro-inflammatory chemokines (CXCL10 and CCL5), with downstream CD8^+^ cytotoxic T-cell infiltration [76]. WEE1 is a checkpoint regulator and its inhibition results in G2/M cell cycle arrest, H2AX phosphorylation and PARP cleavage. In immunocompetent SCLC murine models obtained by conditional loss of *TRP53*, *p130* and *RB1* (called RPP) or *TRP53*, *RB1* and *MYC* (called RPM), they observed that co-treatment with WEE1 inhibitor and PD-L1 antibody induces strong infiltration of CD3^+^, CD8^+^, CD44^+^ effector/memory T-cell and M1 macrophage populations, and complete tumour regression. As summarised in Fig. 1, the molecular mechanism seems to be related to the WEE1 inhibitor induction of micronuclei in SCLC, activation of cGAS, and phosphorylation of downstream proteins (STING, TBK1 and IRF3). RNA-Seq also demonstrated not only enrichment of IFN-α/β pathway but also of IFN-γ, suggesting activation of the STAT1 pathway that mediates the increase in PD-L1 expression on tumour cells, which represents a great therapeutic opportunity [76]. The inhibition of another G2/M kinase, Aurora A, has been recently involved in increased innate immune signalling and, in combination with PD-L1, increased T lymphocyte infiltration, but no cGAS-STING pathway activation [77] (Fig. 1). In a mouse model of ASCL1 subtype, Aurora A inhibition was shown to trap cells in mitosis, causing lower expression of ASCL1 gene and higher MHC-I and interferon target gene expression, suggesting the possibility with these drugs to switch from immuno cold to hot tumours [77].

Zhang et al. focused on *PARP*, which is highly expressed in SCLC, and showed that radiotherapy combined with PARP inhibitor and anti-PD-L1 treatment interestingly prolongs survival in mouse SCLC models and inhibits tumour growth [78]. Specifically, triple-therapy can upregulate PD-L1 on tumour cells by a mechanism related to the increase of its transcription and strongly mediated by STING, as knock-out of *STING* abrogates upregulation of PD-L1. They demonstrated that the combined treatment induces more dsDNA accumulation in the cytoplasm resulting in increased phosphorylation of TBK1 and IRF3 and upregulation of cytokines and chemokines transcription (Fig. 1). The TME is then more infiltrated by CD45^+^ CD3^+^ total cells and CD45^+^ CD3^+^ CD8^+^ cytotoxic T cells, and this more inflamed environment enhances the efficacy of anti-PD-1 therapy on tumour cells [78]. Similarly, another demonstration that SCLC cells can be stimulated for an active innate immunological response was provided by Sen and colleagues, that assessed the effects of prexasertib (CHEK1 inhibitor) and olaparib (PARP inhibitor) [79]. They showed that the drugs induce micronuclei formation and changes in tumour-infiltrating immune cells, although the single treatment did not lead to tumour regression in immunocompetent mice (Fig. 1). They then evaluated the association of each compound with anti-PD-L1 demonstrating a relevant anti-tumour immune response induced by CD3+ and CD8 + T cells, and total tumour regression in animals. They demonstrated activation of the STING pathway in co-treatment, highlighting the phosphorylation of Sting, IRF3, and TBK1, in association with activation of cGAS and induction of chemokine transcription [79]. Because preclinical studies have demonstrated the efficacy of combining DNA damage response inhibition with ICB, clinical trials have been conducted on the combination of PARP1 inhibitors, such as olaparib or niraparib, with anti-PD-L1, such as durvalumab or dorstarlimab [80–82]. Unfortunately, all studies failed to achieve the primary efficacy endpoint, and only a very small number of patients experienced a partial disease control, suggesting that tumour immune phenotypes may be relevant to the response of SCLC to this drug combination.

Cytoplasmatic DNA is a key point to stimulate an innate immune response in cancers cells and, as recently demonstrated, micronuclei are stimulated by a plethora of drugs and compounds, including molecules that target DNA non-canonical structures like G-quadruplexes (G4) [83–85]. Furthermore, it has been shown that sub-cytotoxic concentrations of Top1 poisons can activate the cGAS/STING pathway and immune gene expression in cancer cells, and this process is mediated by the induction of micronuclei in an R-loop-dependent manner [59]. Investigating the molecular mechanism more thoroughly, it has been established that the increase in R-loops induced by Top1 poisons induces damage, and subsequent micronuclei production, following stimulation of the backtracking activity of RNA Polymerase II [86] (Fig. 1). Tested SCLC cell lines (H209, H889, DMS114) have a nonfunctional cGAS/STING pathway [59]. Since *STING* gene is methylated in SCLC cells, resulting in reduced gene expression, it has been evaluated the possibility that demethylating agents or STING overexpression could somehow circumvent the obstacle and induce activation of immune genes. However, the results showed that even in the presence of high cellular STING content, Top1 poisons failed to effectively activate immune genes in SCLC, demonstrating that other players are likely involved in pathway impairment [59]. In H446 cell line, Murayama et al. reported that silencing of DExD/H-box helicase 9 (*DHX9*), a repressor of dsDNA, induces accumulation of dsRNA as well as R-loop and DNA damage-derived cytoplasmic DNA with consequent innate immune response [87] (Fig. 1). Recently, it has also been proposed that in H146 cell line (a STING-low cell line) combined treatment of ATR and Top1 inhibitors can significantly change cytokine and chemokine expression but not in other tested STING-low cell types, suggesting that in this case cellular and molecular heterogeneities among cell lines may play a role in the obtained results [88]. Furthermore, Top2 inhibitors like etoposide are able to stimulate genomic aberration in cancer cells and both Top1 and Top2 inhibitors are able to induce transcription of STING-dependent chemokines like CCL5 and CXCL10 (Fig. 1), disclosing a possible role of these compounds as drugs that can trigger an innate immune response, a key point for immunotherapy efficacy in unresponsive tumours such as SCLC subtypes [89–91].

Since epigenetic regulation plays a role in SCLC transcriptional state of many immune-related genes, different studies propose histone deacetylase (HDAC) inhibitors as a strategy to reactivate STING pathway and immune infiltration in SCLC cell models [63, 92] (Fig. 1). This strategy may also be used to reactivate transposable elements in SCLC that have been shown to correlate with innate immune response and are transcriptionally repressed in SCLC patients [68] (Fig. 1). In melanoma model it has been shown that inhibition of LSD1 is able to induce TEs expression and innate immune response with anti-tumour activity [93]. In addition, high expression levels of ERV LTRs may predict a better survival upon chemotherapy of SCLC patients suggesting that reactivation of patient-specific LTR subfamilies may be a potential strategy for the treatment of immunologically unresponsive SCLC [68]. Inhibition of LSD1 has also been shown to restore MHC-1 expression in SCLC through a transcriptional mechanism and also determines a phenotypic switch from high-NE to low-NE cells and reactivation of NOTCH signalling. All together these data suggest that re-expression of MHC-1 by LSD1 inhibitors can be exploited to stimulate ICB response [94, 95].

Although cancer cells often downregulate STING expression to prevent the innate immune system response, published results show that the pathway can also be activated causing chronic inflammation and cancer progression [96]. Stimulation of NF-kB- rather than IRF3-signature is associated with senescence-associated secretory phenotype (SASP) and tissue destruction [97]. Therefore, we can state that the effects of cGAS/STING pathway potentiation should be analysed according to the molecular features of human cancers.

To overcome the poor immunological response of SCLC and increase immunotherapy efficacy, other signalling pathways could be targeted and therefore alternative approaches have been proposed. The interleukin-15 superagonist N-803 demonstrated to be active in activating NK cells for SCLC cell lysis independently on MHC-I expression and thus activating a response even in immunologically cold tumours [98]. Differently from T cells, NK cells target cancer-presenting ligands without depending on cell surface markers. In SCLC, it has been demonstrated that NK cells are key factor in reducing metastatic spreader, suggesting these cells have a role in immunosurveillance of SCLC dissemination [99]. To test whether NK cell activation could be a useful therapeutic approach to complement immunotherapy, Cish−/− mice, which downregulate negative feedback to IL-15 and thus enhance NK activity, were tested for their response to anti-PD-1 antibody compared to Cish + /+ mice, demonstrating better tumour control than the latter [99]. In the wake of such promising data, Liu et al. developed a CAR-NK system in which delta-like ligand 3 (DLL3), which is a cell surface protein overexpressed in 70% of SCLC cases, was exploited to induce tumour-specific regression in this cell subtype [100]. In SCLC subcutaneous mice models as well as in SCLC pulmonary metastasis, DLL3-CAR-engineered NK-92 cells were able to strongly suppress tumour growth, confirming the growing focus on NK cells as possible alternative strategies in cancer therapy.

## Conclusions

Nowadays lung cancer is the most diagnosed type of tumour and one of the major causes of death in western countries also representing a significant burden and economic loss for the modern society [1]. The advancements accomplished in the last years to understand the mechanisms adopted by the tumour to evade immunosurveillance and, on the contrary, to stimulate immune response in cancer cells, surely improve immunotherapy effectiveness in cancer treatment. Many studies and clinical trials are indeed ongoing to gauge the efficacy of existing and novel combining immunotherapy to treat both NSCLC and SCLC. However, the lack of a valid in vitro and/or in vivo model system for preclinical studies able to evaluate the pharmacological mechanism, kinetics and toxicity of immunotherapy and immunomodulatory agents is a big limit for lung cancer therapy. The investigation of the interplay between cancer and the immune system, as well as the functional immune suppressive mechanisms contributing to immune evasion and/or immunotherapy inefficacy, is crucial for the development of groundbreaking pharmaceutical strategies. In the last years the development of 3D models with the co-culture of tumour and immune system cells such as organoids or tumoroids and the microfluidic-based systems provided the possibility to directly study the patient-derived tumour cells opening new frontiers in the development of personalized medicine for cancer patients [101, 102]. Furthermore, the recent “omics” studies indicate how each patient’s tumour is unique and suggest how individualized treatment could be the future pharmacological perspective for cancer, especially for immunotherapy unresponsive tumours such as SCLC [103]. The immune microenvironment is a dynamic situation and the knowledge of the sophisticated mechanisms that regulate tumour immune escape, immunosuppression or adaptive response to ICB is fundamental to fuel the development of novel strategies and to hope for a better prognosis for SCLC patients.

In this review, we focus on SCLC describing the challenge to classify this tumour using specific master transcription markers, especially considering its heterogeneity and plasticity, to provide new evidence to better understand SCLC subtypes. The current immunotherapy regimens for the treatment of SCLC demonstrate low efficacy and no overall better patients survival, and highlight the immunosuppressive mechanisms of this tumour type. The genetic landscape of the tumour and TME, are key points for understanding the tumour immune response, the reason for ICB failure in some tumours and to hopefully overcome cancer resistance. A key point for improving the efficacy of immunotherapy is the stimulation of innate immunity, and cGAS-STING signalling has for years been considered a therapeutic target to contribute to anti-tumour immunity. Here we gathered evidences showing that in SCLC, the innate immune response can be stimulated by altering the cGAS/STING pathway through micronuclei formation and/or cytoplasmastic DNA sensors regulation, transforming a cold tumour to a potential hot tumour, opening new opportunities for immunotherapy efficacy in SCLC.
